# Comparative 3'UTR Analysis Allows Identification of Regulatory Clusters that Drive Eph/ephrin Expression in Cancer Cell Lines

**DOI:** 10.1371/journal.pone.0002780

**Published:** 2008-07-23

**Authors:** Jennifer Winter, Stefan Roepcke, Sven Krause, Eva-Christina Müller, Albrecht Otto, Martin Vingron, Susann Schweiger

**Affiliations:** 1 Max-Planck Institute for Molecular Genetics, Berlin-Dahlem, Germany; 2 Max-Delbrueck Center of Molecular Medicine, Berlin, Germany; 3 Department of Dermatology, Charité-Hospital, Berlin, Germany; 4 Department of Neuroscience and Pathology, College of Medicine, University of Dundee, Dundee, United Kingdom; Victor Chang Cardiac Research Institute, Australia

## Abstract

Eph receptors are the largest family of receptor tyrosine kinases. Together with their ligands, the ephrins, they fulfill multiple biological functions. Aberrant expression of Ephs/ephrins leading to increased Eph receptor to ephrin ligand ratios is a critical factor in tumorigenesis, indicating that tight regulation of Eph and ephrin expression is essential for normal cell behavior. The 3'-untranslated regions (3'UTRs) of transcripts play an important yet widely underappreciated role in the control of protein expression. Based on the assumption that paralogues of large gene families might exhibit a conserved organization of regulatory elements in their 3'UTRs we applied a novel bioinformatics/molecular biology approach to the 3'UTR sequences of Eph/ephrin transcripts. We identified clusters of motifs consisting of cytoplasmic polyadenylation elements (CPEs), AU-rich elements (AREs) and HuR binding sites. These clusters bind multiple RNA-stabilizing and destabilizing factors, including HuR. Surprisingly, despite its widely accepted role as an mRNA-stabilizing protein, we further show that binding of HuR to these clusters actually destabilizes Eph/ephrin transcripts in tumor cell lines. Consequently, knockdown of HuR greatly modulates expression of multiple Ephs/ephrins at both the mRNA and protein levels. Together our studies suggest that overexpression of HuR as found in many progressive tumors could be causative for disarranged Eph receptor to ephrin ligand ratios leading to a higher degree of tissue invasiveness.

## Introduction

The 3'untranslated regions (3'UTRs) of mRNAs play crucial roles in posttranscriptional regulation of gene expression, for example by modulating mRNA localization [1], [2], [3], stability [4], and translation [5], [6]. Apart from having binding sites for the recently discovered microRNAs, 3'UTRs can harbor motifs that interact with specific RNA-binding proteins. These motifs are normally short-sequence elements whose activity can be influenced by their secondary structure [7]. Therefore, their identification by computer algorithms is difficult and usually produces numerous false positives.

To identify 3'UTR motifs we used a novel approach that is based on two assumptions: first, not only coding regions but also elements within the 3'UTRs that are essential to gene function might be conserved between the paralogues of large gene families; second, mRNAs encoding proteins that functionally interact or fulfill redundant functions might exhibit a conserved organization of regulatory elements in their 3'UTRs. To investigate this, we chose the families of ephrin ligands and Eph receptors. The family of Eph receptors is the largest subfamily of receptor tyrosine kinases. Eph receptors are divided into two subclasses based on their ligand specificities. In general, Eph class A (EphA) receptors bind to glycosylphosphatidylinositol-anchored ephrin A ligands (ephrinA) (with the exception of EphA4), whereas Eph class B (EphB) receptors bind to transmembrane domain-containing ephrin B ligands (ephrinB) [8]. However, more recent data suggest that interactions can also occur across classes [9]. Upon binding to their cognate ephrin ligands, Eph receptors autophosphorylate and activate downstream signaling cascades (forward signaling). Although they do not possess catalytic activity themselves, both classes of ephrin ligands can activate signal transduction pathways after interaction with Eph receptors (reverse signaling) [10].

At the cellular level, signaling through Eph receptors and ephrins leads to either increased adhesion (attraction) or decreased adhesion (repulsion) of the interacting cells. These responses are important in mediating a wide range of biological activities, including angiogenesis, cell segregation, cell attachment, cell morphogenesis, and cell motility [11]. Several of these processes are out of control during tumorigenesis, highlighting a potential critical role for Eph/ephrin signaling in the development of many human cancers. In line with that pathophysiology, several Ephs and ephrins, including EphA1, EphA2, EphA3, EphA4, EphB2, EphB3, and ephrin-A1, are overexpressed in a variety of tumors, and exhibit mostly tumor-promoting properties [11], [12].

Expression patterns of Ephs and ephrins are complex, and their correct expression is essential for the proper function of many of the processes mentioned above. Therefore, a fine-tuned regulation of Eph/ephrin expression both at the transcriptional and posttranscriptional levels would seem to be indispensable. At the transcriptional level, Eph receptors and ephrin ligands have been implicated as downstream targets of many different transcription factors, including homeodomain proteins [13] and the p53 protein family [14], [15], [16], [17]. Much less is known about posttranscriptional regulation. However, there is evidence of involvement of the 3'UTR: upregulation of the EphA2 receptor in axons crossing the midline is mediated by a highly conserved sequence in the transcript's 3'UTR that contains a cytoplasmic polyadenylation element (CPE) [18].

Here we have systematically collected the 3'UTR sequences of mouse Eph receptors and ephrin ligands and screened them for *cis*-acting sequence motifs. We have identified three different types of motifs in several of the Eph/ephrin 3'UTRs: CPEs, AU-rich elements (AREs), and HuR binding sites. Whereas some of these motifs were found to be distributed apparently randomly in the respective 3'UTRs, others were found to be arrayed in clusters which are highly conserved between different vertebrate species. Furthermore, we identified several RNA-binding proteins, including HuR, that interact with these clusters, and we show that EfnA2, EphA2, and EphA4 are direct posttranscriptional targets of HuR in cancer cell lines. Surprisingly, we found that HuR does not act as a stabilizing factor upon expression of these Eph/ephrins, but that it destabilizes the respective messages.

## Results

### The 3'untranslated regions of transcripts of mouse Eph receptors and ephrin ligands

To obtain a complete collection of full-length 3'UTR sequences of mRNAs of Eph receptors and ephrin ligands, we first extracted all available mouse Eph/ephrin sequences from the GenBank database of the NCBI. Only polyadenylated transcripts were considered to be full length, and only such truncating variants that contain both (i) a consensus hexamer sequence near the cleavage site and (ii) a poly(A) tail were included in the analysis. Incomplete transcripts were extended to the poly(A) tail by aligning 3'complete EST data using the software program CAP3 (for accession numbers see Table S1). C-terminal truncated isoforms were not included in the study.

**Figure 1:**
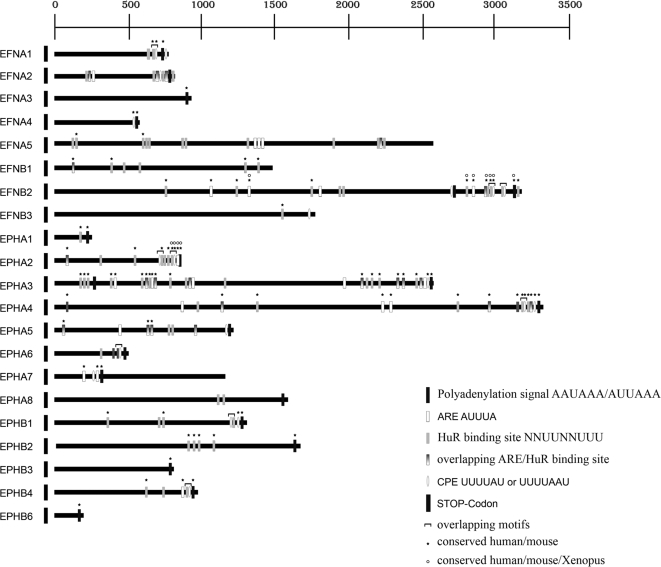
The 3'untranslated regions of Eph receptors and ephrin ligands. Scheme of the 3'UTRs of Eph/ephrins. Putative *cis*-acting sequence motifs are highlighted in different colors, conservation between human/mouse, human/mouse/Xenopus is shown by asterisks/open circles.

Whereas coding regions and exon/intron boundaries [19] are highly conserved between the different members of the Eph receptor and ephrin ligand gene families, the 3'UTRs of transcripts show a high diversity of length and sequence: those of the ephrin ligands vary from 552 bp (EfnA4) to 3171 bp (EfnB2) and those of the Eph receptors from 198 bp (EphB6) to 3310 bp (EphA4) (Fig. 1, Table S1). Moreover, we could not detect any significant conservation at the sequence level, either between the 3'UTRs of the Eph receptor transcripts or those of ephrin ligands (data not shown).

Orthologous conservation between human and mouse varied from 60% to 80%, which is comparable to the genome-wide mean value of 69% [20].

Alternative polyadenylation sites were found in the 3'UTRs of EfnA4, EfnB2, EphA3, and EphA7 (Fig. 1).

### The 3'untranslated regions of transcripts of the Eph receptors and ephrin ligands contain highly conserved *cis*-acting sequence motifs

As outlined above, the sequences of Eph/ephrin 3'UTRs are very poorly conserved between paralogues. However, posttranscriptional regulation often relies on short *cis*-acting sequence motifs, and genes encoding proteins that functionally interact and/or genes belonging to the same gene family might be expected to contain conserved 3'UTR motifs. To detect such elements we scanned the 3'UTRs of the mouse Eph/ephrin transcripts for known *cis*-acting sequence motifs (see http://genereg.molgen.mpg.de/cgi-bin/regEchse/regEchse.pl : motifs that have been included in the search are listed under “select known motif; HuR binding sites were identified using Transterm http://uther.otago.ac.nz/Transterm.html). Remarkably, this led to the identification of three prominent motifs—CPEs, AREs and HuR binding sites—in several of the Eph/ephrin 3'UTRs (Fig. 1). CPEs are translational control units that must reside in close proximity to the poly(A) site to be functional [21]. Therefore, in Figure 1, only those CPEs which fulfill this criterion [0–250 bp distance from poly(A) hexamer] are depicted. HuR interacts with multiple AUUUA repeats [22] and U-rich stretches with high affinity. More recently attempts have been undertaken to more specifically define an HuR binding site [23], [24]. In a series of binding experiments Meisner et al. deduced that the HuR binding site is the 9-mer NNUUNNUUU. Remarkably this motif was found to be present in all validated HuR targets, and a single point mutation within the motif is sufficient to completely destroy HuR binding. In this study the search for HuR binding sites was based on this motif. Both AREs and HuR binding sites were found in all parts of the 3'UTRs. Some of the motifs were found as isolated, non-overlapping elements. In other cases, CPEs were found to overlap with AREs or HuR binding sites, or AREs with HuR binding sites. Of note, all three types of motifs, some in multiple copies, were found together in clusters (Fig. 1, Table S2). These clusters show two distinct localization patterns: located anywhere in the 3'UTR with no obvious preference for a special region (e.g. EphA3, see Fig. 1) or specifically arrayed in the 3'terminal region of the 3'UTR in close proximity to the polyadenylation site (e.g. EphA2, see Fig. 1).

**Figure 2:**
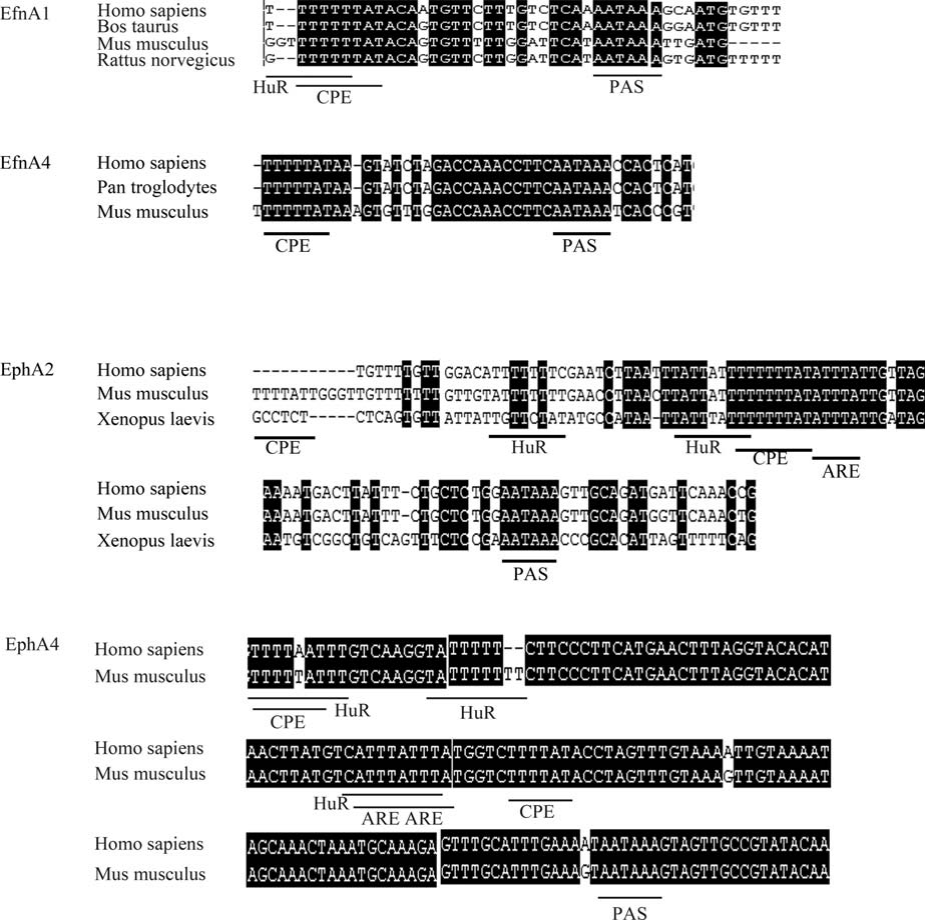
Conservation of 3'terminal parts of Eph/ephrin 3'UTRs containing clustered motifs. Multialignments of representative examples of clustered motifs as found in the 3'terminal parts of the EfnA1, EfnA4, EphA2 and EphA4 3'UTRs. Positions of CPEs, AREs, HuR binding sites as well as the consensus hexamer of the polyadenylation signal are indicated below the alignments.

In orthologous 3'UTRs, *cis*-acting sequence elements with important regulatory functions are expected to be conserved through evolution [24]. For 18 of the 21 mouse Eph/ephrin 3'UTRs investigated in this study, sequences from human orthologues were available, and 44%, 74%, and 72% of CPEs, AREs, and HuR binding sites, respectively, were found to be conserved between the two species (Fig. 1 and 2). Most obvious, almost all of the motifs arrayed in clusters are conserved. Besides mammals, orthologous sequences of the 3'UTRs of EfnB2 and EphA2 from the lower vertebrate *Xenopus laevis* are available. Again, especially those motifs that are arrayed in clusters and located near the poly(A) site were found to be conserved in this species (Fig. 1 and 2).

### Identification of proteins that bind to sequence motifs in the Eph/ephrin 3'UTRs

On the basis of the following criteria, the 3'terminal parts of the EfnA1, EphA2, and EphA4 3'UTRs were selected for further characterization. First, each contains clustered sequence motifs consisting of CPEs and HuR binding sites. EphA2 and EphA4, in addition, contain AREs in these clusters. Therefore, the 3'terminal parts of these 3'UTRs seemed to be good candidates for identification of proteins interacting with the motif clusters. Second, all three motif clusters show a high degree of orthologous conservation in human (Fig. 1). In addition, the EphA2 cluster is highly conserved in *Xenopus laevis* (for EfnA1 and EphA4, no sequence information was available for this species). Third, the 3'terminal region of the EphA2 3'UTR has already been shown to fulfill important regulatory functions [18].

**Figure 3:**
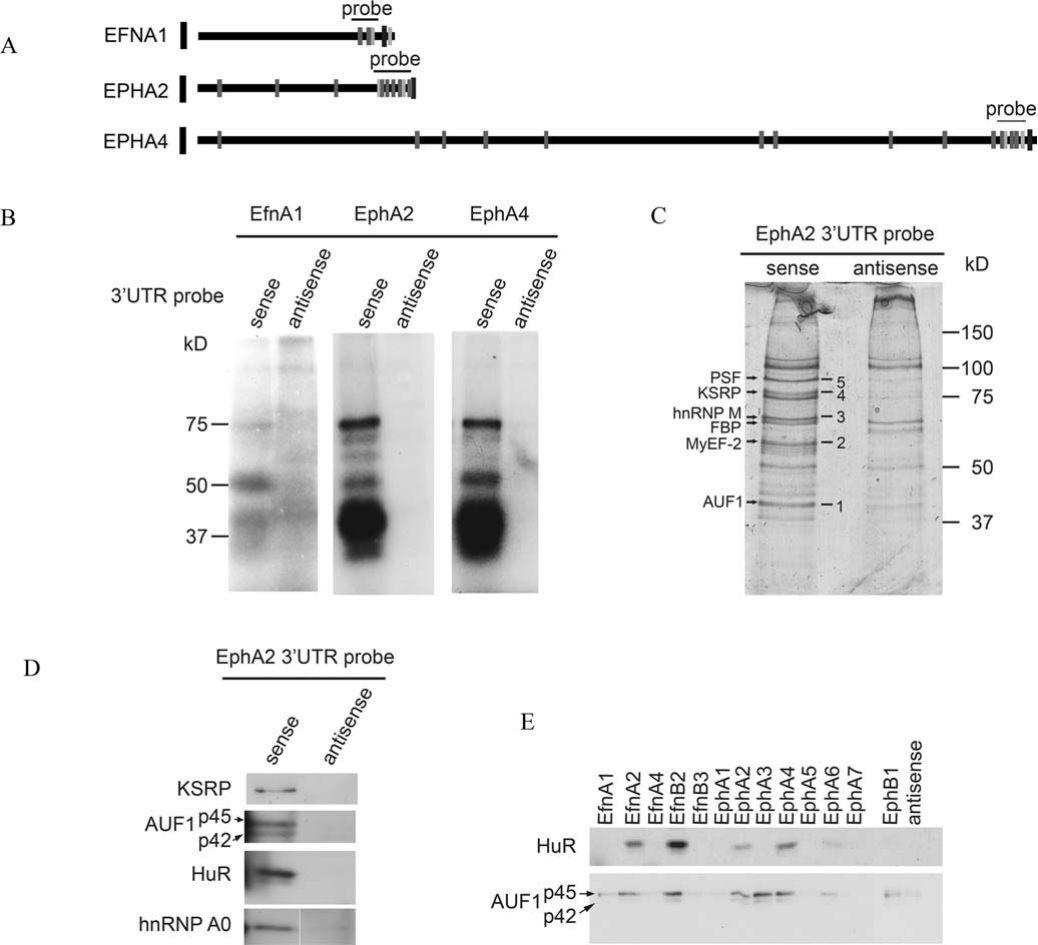
Identification of proteins interacting with clustered motifs harboured in Eph/ephrin 3'UTRs. (A) Scheme of 3'UTRs of EfnA1, EphA2 and EphA4 as depicted in Fig. 1. Probes used for UV-crosslink and RNA-protein pulldown assays are indicated (B) UV-crosslink with protein lysate from mouse brain and radioactively labelled probes corresponding to clustered motifs present in the 3'terminal parts of EfnA1, EphA2 and EphA4 3'UTRs. (C) 10% SDS-gel stained with colloidal Coomassie showing the proteins eluted from streptavidin beads coupled to a biotinylated probe corresponding to the 3'terminal part of the EphA2 3'UTR (left lane) or to the respective antisense sequence (right lane). Differential bands were excised and analyzed by mass spectrometry. Protein identities are given. (D) Western blot analyses of RNA-protein pulldowns with protein lysate from mouse brain and biotinylated probes corresponding to the 3'terminal part of the EphA2 3'UTR (left lane) or to the respective antisense sequence (right lane). KSRP, AUF1, HuR and hnRNP A0 are detected using specific antibodies. (E) Westernblot analyses of RNA-protein pulldowns with protein lysate from mouse brain and biotinylated probes corresponding to the 3'terminal parts of Eph/ephrin 3'UTRs or to the EphA2 antisense sequence. HuR and AUF1 are detected using specific antibodies.

To test for binding of interacting proteins to these motif clusters, radioactively labeled transcripts encompassing the 3'terminal regions of the EfnA1, EphA2, and EphA4 3'UTRs (Fig. 3a) were incubated with lysate from mouse brain and UV-crosslinked. Mouse brain was used in this experiment because several known ARE- and CPE-interacting proteins are highly expressed in this organ. Complexes were resolved by electrophoresis through SDS acrylamide gels, and the dried gels were exposed to X-ray film. Interestingly, this method not only identified several proteins that had bound to the motif clusters of all three 3'terminal regions, but also revealed an overlapping banding pattern (Fig. 3b): proteins of 30–45, 50, and 75 kD bound to all three sequences, although the affinity to the 3'terminal regions of the EphA2 and EphA4 3'UTRs seemed much higher than to that of the EfnA1 3'UTR. Proteins of ∼60 and 70 kD were exclusively detectable in the EphA2 and EphA4 lanes (Fig. 3b). Identities of the interacting proteins were determined in an RNA–protein pulldown assay with subsequent analysis of the eluted fraction by mass spectrometry. Biotin-labeled *in*-*vitro*-transcribed transcripts corresponding to the 3'terminal region of the EphA2 3'UTR were incubated with lysate from mouse brain and pulled down with streptavidin magnetic beads. Transcripts in antisense orientation to the chosen region were used in a negative control experiment. Complexes were resolved by SDS-PAGE, stained with colloidal Coomassie, and bands specifically enriched in the sample obtained with the sense transcripts were analyzed by electrospray ionization (ESI) mass spectrometry. Interestingly, in addition to several proteins that are known to be involved in transcription and mRNA splicing—myelin expression factor 2 (MyEF-2); heterogeneous nuclear ribonucleoprotein M (hnRNP M); far upstream binding protein 1 (FUSE-binding protein 1, FBP); splicing factor, proline- and glutamine-rich (PSF)—we identified AUF1 and KSRP, two ARE-binding proteins (ARE-BPs) that are known regulators of mRNA stability (Fig. 3c, Table 1). To confirm the interaction we repeated the RNA–protein pulldown assay. This time, however, RNA–protein complexes were transferred to PVDF membranes and interacting proteins were detected with specific antibodies. As expected, KSRP bound to the RNA probe corresponding to the EphA2 3'UTR terminal region but not to the antisense negative control (Fig. 3d). Interestingly, concerning AUF1, we could only detect binding of the p45 and p42 isoforms but not the p40 and p37 isoforms. Besides ARE and CPE-motifs, the 3'terminal part of the EphA2 3'UTR harbors a HuR binding site of the consensus NNUUNNUUU. However, HuR was not identified as an interacting protein in the mass spectrometry analysis. To test directly for the binding of HuR we incubated the Western blot membrane with an antibody directed against endogenous HuR protein. As expected, HuR was found to specifically interact with the 3'terminal region of the EphA2 3'UTR.

**Table 1:** List of proteins that were found to be interacting with the 3'terminal part of the EphA2 3'UTR and their most important functions.

Protein	Accession number	Function	References
Heterogenous nuclear ribonucleoprotein D0 (HNRPD, AUF1)	Q60668	RNA/DNA binding, ARE-binding, enhancement of mRNA decay, telomere elongation, transcription	[48], [49], [50], [51], [52]
Myelin expression factor 2 (MyEF-2)	Q8C854	Transcriptional repressor, suppresses transcription of mouse myelin basic protein gene	[53], [54]
Heterogenous nuclear ribonucleoprotein M (hnRNP M)	Q9D0E1	RNA binding, splicing, carcinoembryonic antigen receptor	[55], [56], [57], [58]
Far upstream element-binding protein 1 (FUSE-binding protein 1, FBP)	Q91WJ8	Transcription factor, regulates MYC expression	[59], [60]
Far upstream element-binding protein 2 (KSRP)	Q99PF5	RNA/DNA binding, ARE-binding, alternative splicing, degradation of ARE-containing mRNAs, activation of gene expression	[61], [62], [63], [64]
Splicing factor, praline- and glutamine-rich (PSF)	Q8VIJ6	RNA/DNA binding, splicing, homologous DNA pairing, transcriptional regulation	[65], [66], [67]

Based on size similarities of 30–45 kD, the UV-crosslinking assay suggested binding of the same proteins to the 3'terminal parts of the EfnA1, EphA2, and EphA4 3'UTRs (Fig. 3b). This might indicate that expression of several of the Eph receptors and ephrins is regulated by a shared mechanism. To prove that identical proteins bind to the motif clusters identified in several Eph/ephrin 3'UTRs, we performed additional RNA–protein pulldown assays with *in*-*vitro*-transcribed and biotinylated 3' terminal parts of all the Eph/ephrin 3'UTRs that contain AREs, CPEs and/or HuR binding sites (Table 2). In addition, we used the 3' terminal part of an Eph 3'UTR that does not contain such a motif (EphA1; the only motif identified in the 3'UTR of this transcript is an HuR binding site located outside the 3' terminal part). Again, the complexes were transferred to PVDF membranes, and these were incubated with antibodies detecting the 32 kD HuR protein and the four AUF1 isoforms, p37, p40, p42, and p45, respectively. To our surprise we found that both HuR and AUF1 p42/p45 bound to some but not all of the transcripts (Fig. 3e), and the binding patterns of both proteins were only partially overlapping: both HuR and AUF1 interacted with the 3' terminal regions of the EfnA2, EfnB2, EphA2, EphA4, and EphA6 3'UTRs, but only AUF1 bound to the 3' terminal regions of EfnA1, EphA3, and EphB1 3'UTRs (Fig. 3e). No binding of either AUF1 or HuR was seen with the 3' terminal region of EphA1, which does not contain a motif.

**Table 2:** Motifs in biotinylated probes used for RNA-protein pulldown assay and binding patterns of AUF1 (p42/p45) and HuR.

	CPE	ARE	Bound by AUF1 Fig. 3d	HuR binding site (total)	HuR binding site (single strand conformation)	Bound by HuR Fig. 3d
EfnA1	1	−	+	2	−	−
EfnA2	1	2	++	4	2	++
EfnA4	1	−	−	−	−	−
EfnB2	2	−	++	3	1	++
EfnB3	1	−	−	1	−	−
EphA1	−	−	−	−	−	−
EphA2	2	1	++	3	1	+
EphA3	1	1	++	−	−	−
EphA4	2	1	++	3	1	++
EphA5	1	−	−	−	−	−
EphA6	1	−	+	1	1	+
EphA7	1	1	−	−	−	−
EphB1	2	−	+	1	−	−

A closer look at the composition of the different 3'UTR terminal parts revealed that AUF1 had bound to all ARE-containing transcripts except EphA7, but also to four transcripts that did not contain an ARE (EfnA1, EfnB2, EphA6, EphB1). For binding of HuR, presence of the consensus HuR binding site was essential but not sufficient: we could not detect binding of HuR to any of the transcripts lacking this site; however, of the eight transcripts containing this site, HuR bound to EfnA2, EfnB2, EphA2, EphA4, and EphA6 but not to EfnA1, EfnB3, and EphB1.

**Figure 4:**
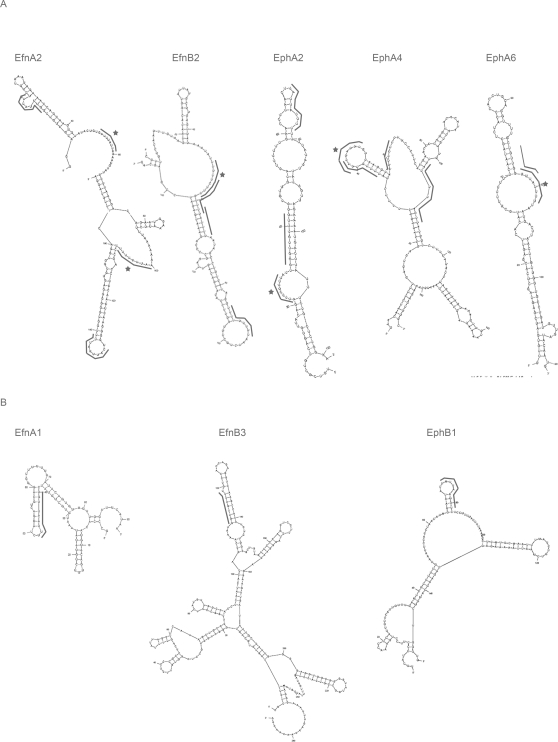
Secondary structures of Eph/ephrin 3'terminal parts. Shown are the secondary structures of the probes used in UV-crosslinking and RNA-protein pulldown experiments that correspond to the 3'terminal parts of Eph/ephrin 3'UTRs as predicted by Mfold. (A) Probes with HuR binding sites located in single stranded regions (asterisks) that showed high affinity binding of HuR. (B) Probes where HuR binding sites were predicted to be harboured in double stranded regions.

Because HuR binds to single-stranded RNA, it was proposed that the HuR binding site might have to assume a particular conformation to undergo high-affinity HuR binding [24]. Therefore, we used the Mfold program to predict secondary structures in the 3'UTR probes that we had used in the RNA–protein pulldown assay. In agreement with the results of Meisner et al. (2004), we found that HuR had interacted only with those transcripts in which the HuR binding sites were predicted to be located in single-stranded loops (Fig. 4a). The HuR binding sites of the remaining transcripts were all (at least partially) located in double-stranded regions (Fig. 4b) and therefore possibly inaccessible to HuR.

The secondary structure of a relatively short partial transcript may differ greatly from the structure of the whole 3'UTR and therefore might not reflect the conformation *in vivo*. To gain insight into the accessibility of the HuR binding sites harbored in the Eph/ephrin 3'UTRs we used the Mfold algorithm to predict the secondary structures of the entire 3'UTRs. Interestingly, apart from EfnB3 and EphA1, all murine Eph/ephrin 3'UTRs contain at least one HuR binding site predicted to be part of a single-stranded region in or outside of the described motif clusters, suggesting a particularly important role of HuR in the regulation of Eph/ephrin expression (Fig. S1).

### HuR regulates expression of Eph/ephrins in cancer cell lines

**Figure 5:**
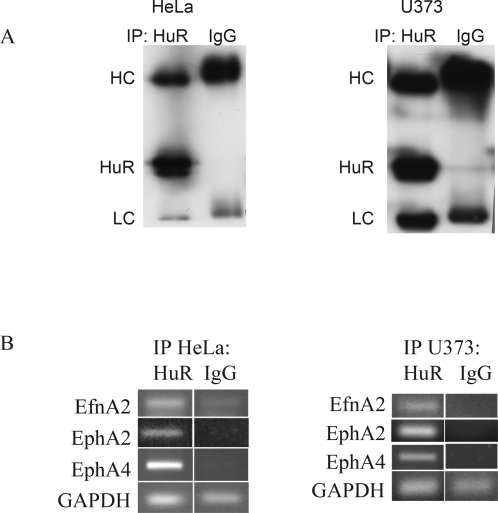
*In vivo* binding of HuR to Eph/ephrin mRNAs. (A) Immunoprecipitation of endogenous HuR. HeLa or U373 cell lysates were immunoprecipitated with anti-HuR antibody or with unspecific IgGs. Immunoprecipitates were loaded on 10% SDS-Pages and analysed by westernblot with anti-HuR antibody; HC, heavy chain; LC, light chain (B) RT-PCR after RNA-extraction from the HuR and IgG immunoprecipitates with primers specific for EfnA2, EphA2, EphA4 and GAPDH.

Abnormal expression of Eph/ephrins has been reported in various cancers, and is associated with poor prognosis and advanced stages of malignancy [11], [25]. While much effort has been put into the elucidation of transcriptional regulation of Eph/ephrins in cancer cell lines and cancerous tissues, posttranscriptional regulation has been neglected. Seventy-two percent of the HuR binding sites of mouse Eph/ephrin 3'UTRs are conserved in human (see above). To test if our findings in mouse cells also apply to human cells we immunoprecipitated HuR from the cervical carcinoma cell line HeLa and the astrocytoma/glioblastoma cell line U373MG (Fig. 5a) using an anti-HuR antibody, and performed RT-PCR on the immunoprecipitate with primers specific for EfnA2, EphA2, and EphA4, which are three mRNAs known to be highly expressed in various cancer types [11]. As negative control we performed the same procedure with nonspecific immunoglobulins instead of anti-HuR antibody (Fig. 5a and b), and as negative control for RT-PCR we performed the cDNA-synthesis step without reverse transcriptase (data not shown). Compared to the negative IgG control, there was clear enrichment of EfnA2, EphA2, and EphA4 messages in the immunoprecipitates of both cell lines (Fig. 5b) indicating that HuR had bound to the Eph/ephrin mRNAs *in vivo*. Of note, non-target GAPDH message could also be amplified, albeit less efficiently and to the same extent in both IP groups; this finding has been described before [23], and verified the use of equal amounts of input material.

**Figure 6:**
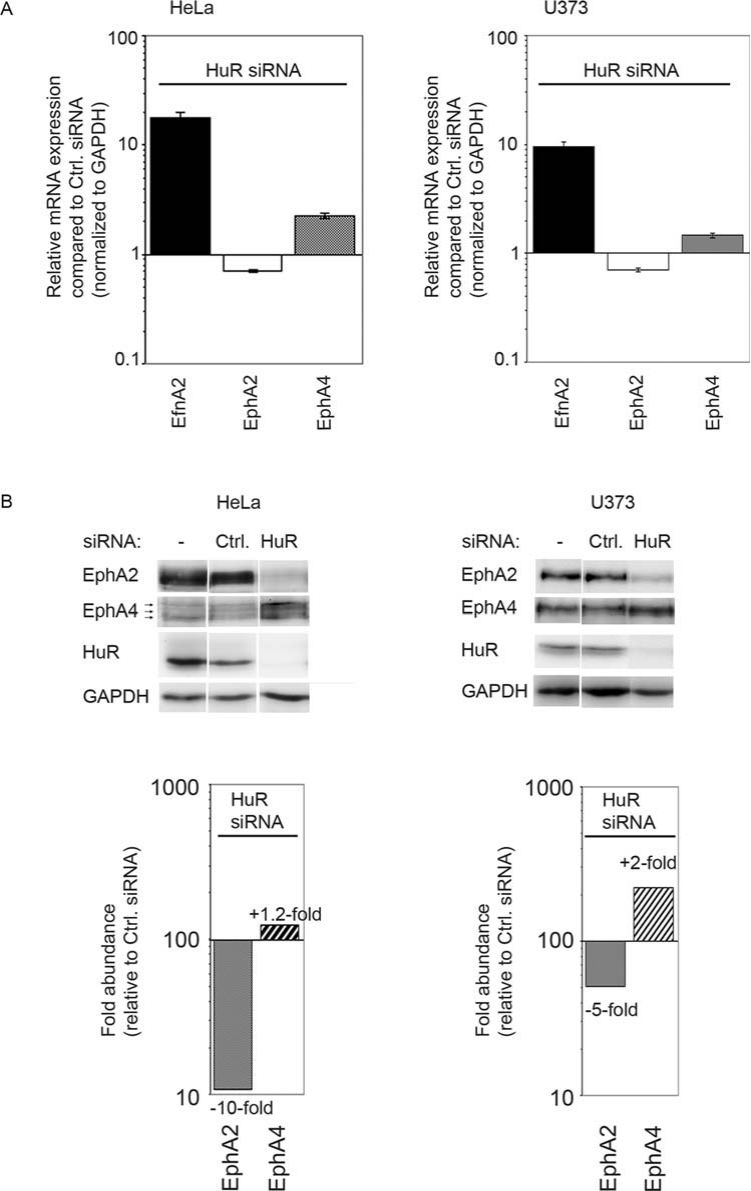
HuR regulates expression of Eph/ephrins. HuR was knocked down with a specific HuR RNAi oligonucleotide in HeLa and U373 cells (A) mRNA expression of EfnA2, EphA2 and EphA4 72 hours after transfection of a specific HuR RNAi oligo was compared to transfection of a non-silencing control oligo as measured by Real Time RT-PCR. Ratios between expression levels after HuR knockdown and tranfection of non-silencing control oligonucleotodides are shown. Values were normalized to GAPDH. (B) Westernblot analyses of protein expression of EphA2, EphA4 (three isoforms, see arrows), HuR and GAPDH 72 hours after knockdown of HuR (upper panel, lane 3) with specific antibodies in comparison to a mock-control (lane 1) as well as a non-silencing siRNA control (lane 2). Quantification of bands (Against GAPDH) was performed using the Image Quant software 5.2 (lower panel). Fold increase or decrease of expression are depicted.

In a further series of experiments we tested whether HuR posttranscriptionally regulates Eph/ephrin expression. We knocked down HuR in HeLa and U373MG cells using two different HuR-specific siRNA oligonucleotides (Fig. 6a and data not shown) and detected EfnA2, EphA2, and EphA4 mRNAs by Real-Time RT-PCR 72 h after transfection. HuR has been reported as an mRNA stabilizing protein, suggesting that its knock-down would lead to a reduction of Eph/ephrin mRNA expression. However, whereas the amount of EphA2 mRNA did decrease after knock-down of HuR, to our surprise the EfnA2 and EphA4 mRNA levels increased significantly (Fig. 6a). To test whether the changes observed at the RNA level reflect the situation at the protein level we loaded equal amounts of protein lysates from HeLa and U373 cells after HuR knock-down and performed Western blot analysis with antibodies detecting EphA2 and EphA4 (Fig. 6b). In confirmation of the Real-Time PCR experiments we found that expression of EphA2 protein was drastically reduced by 10-fold in HeLa and 5-fold in U373 cells after knock-down of HuR, whereas expression of EphA4 was increased by 1.2-fold and 2-fold, respectively (Fig. 6b). Control siRNA oligonucleotides did not have a significant effect on EphA2 or EphA4 protein expression when compared to a mock-transfected control. Of note, the commercially available EphA4 antibody used in this experiment detects three different bands in HeLa cells (Fig. 6b), all of which were upregulated after HuR knock-down. Two of these bands may correspond to two different EphA4 isoforms with a size difference of 37 amino acids that are listed in the Genome Browser. The third band may represent a HeLa-specific isoform that has not yet been detected in any of the conventional databases.

**Figure 7:**
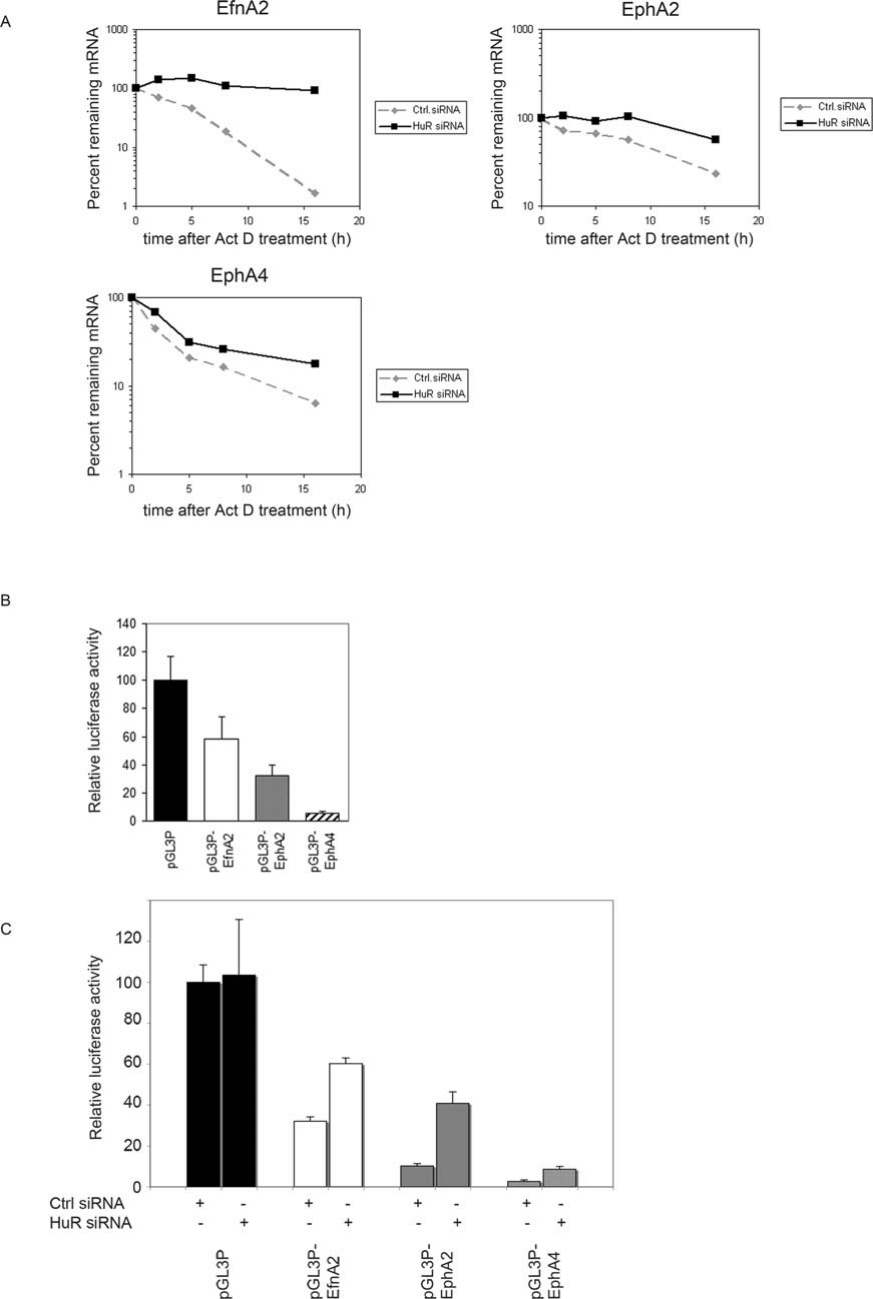
HuR is a direct regulator of Eph/ephrin mRNA stability. (A) 48 hours after transfection of a specific HuR RNAi oligonucleotide or a control oligonucleotide the half-lives of EfnA2, EphA2 and EphA4 mRNAs were assessed by using 5 µg/ml Actinomycin D; mRNA half-lives were measured by Real Time RT-PCR, mRNA expression was normalized to GAPDH. (B) 3'terminal parts of EfnA2, EphA2 and EphA4 3'UTRs were cloned in pGL3 promoter vector and transfected into HeLa cells. 30 hours after transfection cells were harvested and firefly luciferase activity was measured. For normalization a Renilla luciferase plasmid (pRL) was co-transfected. (C) 24 hours after transfection of a specific HuR RNAi oligonucleotide HeLa cells were transfected with pGL3 promoter constructs as described in (B), and 48 hours later firefly luciferase activity was measured, and normalized to the co-transfected pRL plasmid.

To distinguish between indirect transcriptional effects and direct mRNA stabilization caused by the knock-down of HuR, we blocked transcription with actinomycin D 48 h after transfection of HuR siRNA oligos or control oligos, and measured the mRNA half-lives of EfnA2, EphA2, and EphA4 by Real-Time RT-PCR. Surprisingly, we found that knock-down of HuR increased the half-lives of all Eph/ephrin mRNAs tested. This suggests that HuR destabilizes these mRNAs (Fig. 7a), and that the decrease in EphA2 message detected after knock-down of HuR may be caused by secondary inhibition of EphA2 transcription.

To further confirm that the observed effects are mediated by *cis*-acting sequences harbored in the 3'terminal parts of the EfnA2, EphA2, and EphA4 3'UTRs, pGL3P reporter plasmids carrying these sequences (including the HuR binding sites) downstream of the firefly luciferase cDNA were transfected into HeLa cells. For normalization, the pRL vector containing the *Renilla* luciferase cDNA was co-transfected. pGL3P constructs containing the EfnA2, EphA2 or EphA4 3'terminal sequences produced significantly less firefly luciferase activity than the pGL3P vector without 3'UTR inserts, confirming a destabilizing function for the 3'terminal parts of these 3'UTRs (Fig. 7b). After knock-down of HuR with a specific RNAi oligo this destabilizing effect could be partially rescued, which further suggests a destabilizing function for the HuR protein (Fig. 7c). Knock-down of HuR with an RNAi oligo targeting a different region of the HuR mRNA showed comparable results (data not shown). Off-target effects were excluded by transfecting a pGL3P vector without HuR binding sites.

## Discussion

In this study we used a bioinformatics/molecular biology approach to screen the 3'UTRs of transcripts of Eph receptors and ephrin ligands for putative *cis*-acting sequence motifs and to characterize their biological function.

We show that several of the Eph/ephrin 3'UTRs, although exhibiting substantial divergence between paralogs in terms of length and sequence, contain evolutionarily conserved motif clusters composed of overlapping CPEs, AREs, and HuR binding sites. We identified HuR and several other proteins involved in mRNA stability control as interaction partners of these clusters. Switching from mice to human cancer cell lines, we also obtained evidence that expression of Eph/ephrins is posttranscriptionally regulated through interaction of the conserved sequence clusters and HuR, suggesting a link between misregulation of Eph/ephrin expression in cancer and overexpression of HuR.

### The 3'untranslated regions of transcripts of mouse Eph receptors and ephrin ligands

A hallmark of members of the Eph/ephrin families is their expansion of numbers during the course of evolution, due to multiple duplication events [19]. The respective coding regions and exon/intron boundaries are highly conserved among orthologues and paralogues, which is reflected by the redundant functions that Eph/ephrins can fulfill and by the ability of Eph receptors to interact with multiple ephrins. Moreover, the 3'UTRs of Eph/ephrin orthologues are highly conserved between human and mouse (up to 80%), which indicates strong selection pressure and perhaps conservation of distinct expression profiles. In contrast, the 3'UTRs of Eph/ephrin paralogues show a high degree of structural divergence, which may have enabled Ephs/ephrins to acquire diverse expression profiles (cellular or subcellular), as has been suggested for other conserved paralogues [26]. Despite their poor overall conservation, the 3'UTRs of 7 of 8 ephrin ligands and 11 of 13 Eph receptors contain CPEs, AREs, and/or HuR binding sites. The grouping of several of these motifs into evolutionarily conserved clusters further emphasizes their functional importance. However, because neither the position nor composition nor distribution of these motifs is conserved between paralogous Eph/ephrins, the clusters may have arisen independently in evolution. Such convergent evolution has been described for regulatory elements of other genes coding for proteins that functionally interact [27], [28], [29], [30]. This is in line with our observation that conserved motif clusters are present in functionally interacting Eph/ephrins. For example, transcripts of the EphA2 receptor and its ligand, ephrin-A1, both contain conserved clustered motifs, as do the EphA4 receptor and its ligands, ephrin-A1 and ephrin-B2. Several clustered motifs are located close to poly(A) sites, which may reflect a function for these clusters in polyadenylation, as discussed below.

We identified the ARE-binding proteins AUF1 (p42/p45), KSRP, hnRNP A0, and HuR as interaction partners of the 3'terminal ARE/CPE/HuR clusters. However, we were not able to show binding of the CPE-interacting factor, cytoplasmic polyadenylation element binding protein (CPEB), even though the RNA–protein pulldown assays were done with lysate from mouse brain, a tissue in which CPEB is strongly expressed and functional [31], [32], [33]. Whereas we cannot exclude the possibility that we could not detect binding of CPEB to Eph/ephrin CPEs for technical reasons, it might also be that either CPEB is not an interactor of these CPEs or it was outcompeted by other proteins that interacted with the AREs or HuR binding sites overlapping with or located in close proximity to the CPEs. Future studies employing direct binding assays should help answer this question.

According to their primary structure, AREs are subdivided into three different classes: class I AREs consist of several dispersed copies of the AUUUA motif, class II AREs contain at least two overlapping UUAUUUA(U/A)(U/A) nonamers, and class III AREs are purely U-rich regions without an AUUUA motif [22], [34]. Initially, HuR had been described as interacting with all three classes summarized in [34]. Since then several groups have tried to identify a more specific HuR binding site [23], [24]. The binding pattern of HuR to Eph/ephrin 3'UTR probes used in our study suggests binding of HuR to a single-stranded consensus motif (NNUUNNUUU), which had been reported previously [24].

### HuR regulates expression of Eph/ephrins in cancer cell lines

The human ELAV-like protein HuR is a well-characterized ARE-binding protein that has been shown to stabilize a variety of mRNA targets, including cyclooxygenase-2, cyclin A, cyclin B1, and others [22]. However, HuR has also been shown to modulate translation of a number of targets [35], [36], [37]. Finally, it was demonstrated recently that mammalian Hu proteins regulate polyadenylation by blocking poly(A) sites containing U-rich sequences [38], and the *Drosophila* homolog of HuR, ELAV, was shown to inhibit 3'-end processing within the non-neuronal exon of the *ewg* pre-mRNA to promote neural splicing [39], [40]. It is widely accepted that HuR stabilizes transcripts by hindering destabilizing factors from binding to the same or overlapping sites. In the case of Eph/ephrins, where we see a destabilizing effect of HuR, it would be interesting to investigate whether a block of polyadenylation may contribute to destabilization of Eph/ephrin mRNAs by HuR. Although speculative, this hypothesis is supported by the fact that several of the HuR binding sites found in Eph/ephrin 3'UTRs are located in conserved clusters near the poly(A) site. Moreover, the blocking effect observed by Zhu et al. (2007) was mediated by binding of HuR to U-rich sequences located immediately upstream of the cleavage site. It seems reasonable to assume that blocking polyadenylation has consequences for the stability of the respective transcripts because polyadenylation can enhance RNA stability. In this context it is plausible that the position of HuR binding sites in a given 3'UTR has a significant influence on the functional outcome.

Whereas the destabilizing function HuR exerts on the EfnA2, EphA2, and EphA4 transcripts was in accordance with an increase in the RNA level for two of these messages after HuR knock-down, EphA2 showed an unexpected decrease. This may be due to secondary transcriptional effects, which could be mediated by binding of HuR to the mRNAs encoding transcription factors that regulate EphA2 expression. Good candidates would be p53 or c-myc, which both (i) regulate EphA2 transcription (p53 positively and c-myc negatively), and (ii) are targets of HuR [17], [41]. Moreover, we cannot exclude the possibility that the potential use of alternative poly(A) sites that, although as yet undetected, might be harboured in one or the other Eph/ephrin 3'UTR could have contributed to the observed differences in Eph/ephrin expression after HuR knockdown. The use of recently developed HuR inhibitors [42] should help to clarify this issue and make it possible to separate short-term effects directly related to the stability of the target mRNA from long-term secondary effects.

Overexpression of Eph/ephrins has been shown in several tumor types and seems to be a prognostic marker that correlates with tumor malignancy [11]. The highest levels of EphA2, for example, are present on the most invasive tumor cells. It has been suggested that not only overexpression of single Eph receptors contribute to tissue invasiveness but also a disturbed receptor/ligand density ratio. Also HuR was shown to have oncogenic potential and its overexpression seems to correlate with advanced stages of tumor malignancy [25]. As we show here HuR heavily influences the expression of many Ephs and ephrins either by direct effects on the stability of the respective transcripts and/or by secondary effects on the transcriptional levels. Therefore, it seems plausible that overexpression of HuR in tumors completely disarranges the expression patterns of multiple Ephs/ephrins leading to a disequilibrium of receptor to ligand ratios and consequently to enhanced invasiveness. In the future it will be interesting to analyze a set of different tumor tissues for the expression of both Eph/ephrins and HuR and correlate these data with tumor phenotype and patient outcome.

## Methods

### Antibodies and siRNAs

Anti-KSRP antibody was a kind gift of Douglas Black. Commercially available antibodies were used for detection of HuR (Santa Cruz Biotech., 3A2), hnRNPA0, GAPDH (Santa Cruz Biotech.) and AUF1 (Upstate Biotech.). For knockdown of HuR validated siRNAs targeting two different regions of the coding region were used (Qiagen, Hs_ELAVL1_1_HP and Hs_ELAVL1_11_HP). As control a nonsilencing siRNA was used (target sequence: AATTCTCCGAACGTGTCACGT).

### Constructs

643–745, 646–841, 721–865, and 3101–3309 3'UTRs of EfnA1, EfnA2, EphA2, and EphA4, respectively, were cloned in the pGL3-Promoter vector (Promega) using the XbaI site located downstream of the firefly luciferase sequence (position 1934).

### Cell culture, RNA interference, Real Time RT-PCR and Luciferase assays

Human cervical carcinoma HeLa cells were cultured in DMEM (Lonza) supplemented with 10% fetal bovine serum, glutamine and antibiotics. U373MG cells were cultured in EMEM, supplemented with 10% fetal bovine serum, glutamine, nonessential amino acids, sodium pyruvate, and antibiotics. For siRNA analyses, cells were seeded at a density of 1×10^4^ cells/well (24-well plate) or at a density of 1×10^5^ (6-well plate) and transfected with 1 µl and 5 µl of 20 µM siRNA/well, respectively, using Oligofectamine™ in OptiMEM. After 72 h, cells were harvested and the efficiency of the knock-down was tested by Western blot with specific antibodies. For mRNA stability assays, cells were treated with 5 µg/ml actinomycin D 48 h after transfection.

For Real Time RT-PCR, RNA was isolated using an RNeasy Mini Kit (Qiagen). cDNA was synthesized using a TaqMan reverse transcription reagents kit (Applied Biosystems), and real-time PCR was carried out using a SYBRGreen PCR master mix (Applied Biosystems) according to the manufacturer's instructions with an ABI 7900HT cycler under the following conditions: 50°C for 2 min; 95°C for 10 min; 95°C for 15 s, 60°C for 1 min for 40 cycles; and 95°C for 15 min, 60°C for 15 min, 95°C for 15 min for the dissociation stage. Primers used are listed (Table S3).

For Luciferase assays, cells were transfected either 24 h after seeding in 24-well plates or 24 h after transfecting siRNAs with 475 ng of constructs cloned in pGL3-promoter vector and 95 pg of pRL control vector using Lipofectamine2000 (Invitrogen). Luciferase activity was measured 30 h or 48 h later using a Dual-Luciferase Reporter Assay System (Promega).

### *In vitro* transcription

^32^P-labeled cRNAs or biotin-labeled cRNAs corresponding to the sense 643–734, 646–815, 447–528, 2935–3080, 1461–1727, 181–293, 721–841, 2497–2594, 3101–3271, 1129–1225, 401–521, 247–369, and 1201–1351 3'UTRs of EfnA1, EfnA2, EfnA4, EfnB2, EfnB3, EphA1, EphA2, EphA3, EphA4, EphA5, EphA6, EphA7, and EphB1, respectively, and to the corresponding antisense 3'UTRs of EfnA1, EphA2, and EphA4 were produced using purified PCR-amplified cDNA, which included the T7 Polymerase promoter sequence, and T7 polymerase (Promega) according to the manufacturer's procedure. Primer sequences are given in Table S4. *In*-*vitro*-transcribed probes were DNAse-treated and ethanol-precipitated.

### UV-crosslinking assay

Brains from adult NMRI mice were frozen in liquid nitrogen, pulverized using a mortar and pestle, resuspended in TKM buffer (20 mM Tris [pH 7.5], 150 mM KCl, 5 mM MgCl2) supplemented with 1% NP40, 1 mM DTT, and complete protease inhibitor cocktail (Roche), lysed with ultrasound, and centrifuged at 12,000×g for 15 min at 4°C. Reaction mixtures containing 20 µg of protein lysate in reaction buffer (5.2 mM HEPES [pH 7.9], 50 mM KCl, 10 mM DTT, 5 mg/ml heparin, 1% glycerol, 40 µg/ml yeast tRNA) and 250,000 cpm of radiolabeled probe were incubated for 10 min at room temperature, UV-crosslinked for 10 min in a UV Stratalinker 1800 (Stratagene) and digested with 1 U each of RNAse A and RNAse T1 for 15 min at 37°C. Complexes were resolved by electrophoresis through SDS–10% acrylamide gels, after denaturation at 95°C for 5 min. Gels were dried and exposed to X-ray film.

### RNA–protein pulldown

To obtain pure protein lysate, mouse brains were treated as described above. After the first centrifugation at 12,000×g, ultracentrifugation was performed at 100,000×g for 1 h at 4°C. Reaction mixtures containing 6 mg (for Coomassie staining) or 200 µg (for Western blot analysis) of protein lysate in TKM buffer supplemented with 1% NP40, 1 mM DTT, complete protease inhibitor cocktail (Roche), 100 U of RNasin (Promega), and 40 µg (for Coomassie staining) or 3 µg (for Western blot analysis) of biotin-labeled probe were incubated for 1 h at 4°C, followed by the addition of streptavidin magnetic beads and incubation for 2 h at 4°C. After washing and denaturation at 95°C for 5 min, proteins were resolved by electrophoresis through SDS-10% acrylamide gels. Gels were either stained with colloidal Coomassie and analyzed by mass spectrometry or blotted on PVDF membranes and Western blot analyses performed with antibodies directed against the respective proteins.

### Mass spectrometry

The peptide mixture was identified by chromatographic separation on an LC Packings 75 _m PepMap C18 column (Dionex, Idstein, Germany) using a capillary liquid chromatography (CapLC) system delivering a gradient to formic acid (0.1%) and acetonitrile (80%). Eluted peptides were ionized by electrospray ionization on a Q-TOF hybrid mass spectrometer (Micromass, Manchester, UK). The mass spectral data were processed into peak lists containing the *m*/*z* value, charge state of the parent ion, fragment ion masses and intensities, and correlated with the SwissProt database using Mascot software [43].

### Immunoprecipitation and RT-PCR

For immunoprecipitation of endogenous mRNPs the protocol published by [44] was used with the following modifications: For immunoprecipitation of HuR the protein lysates were precleared for 1 h at 4°C with protein-G Agarose. Complexes were immunoprecipitated for 2 h at room temperature using 10 µg of HuR antibody or the corresponding amount of IgG, followed by incubation for 1 h at room temperature in the presence of protein-G agarose. RNA was extracted using an RNeasy Mini Kit (Qiagen).

For RT-PCR, 50% of the RNA isolated from the immunoprecipitates (IPs) was reverse-transcribed using random hexamers, and SSIII reverse transcriptase (RT) (Invitrogen). The other 50% served as negative controls (no RT in the reaction mixture). 1–3 µl of the resulting cDNA was used for PCR amplification using 95°C (5 min) followed by 30–35 cycles 95°C (30 s), 55°C (1 min), and 72°C (1 min), then 5 min at 72°C (primer sequences are given in Table S3).

### Bioinformatics analyses

To obtain the complete sequences of the 3'UTRs we first extracted all available sequences from the GenBank database of the NCBI. Transcripts were considered complete if they were polyadenylated at their 3'ends. Incomplete transcripts were extended to the poly(A) tail by aligning 3'-complete EST data using the software program CAP3 (http://pbil.univ-lyon1.fr/cap3.php) [45].

Eph and ephrin 3'UTRs were scanned for known binding motifs for RNA-binding proteins using BioPerl [30]. For detection of G quartets, CPEs and AREs we used the minimal elements WGG WGG WGG WGG, TTTTAT, and ATTTA which have been shown to suffice for binding of interacting proteins [10] [26]. For detection of HuR binding sites, Eph and ephrin 3'UTRs were scanned for the sequence motif NNUUNNUUU [24] using TransTerm (http://uther.otago.ac.nz/Transterm.html) [46]. RNA secondary structures were predicted using Mfold [47].

## Supporting Information

Table S1 Lengths of Eph/ephrin 3'UTRs and accession numbers of Eph/ephrin mRNAs
(0.04 MB DOC)

Table S2 Positions of CPEs, AREs, HuR binding sites and poly(A) hexamers in the Eph/ephrin 3'UTRs
(0.05 MB DOC)

Table S3 Primers used for Real Time RT-PCR
(0.03 MB DOC)

Table S4 Primers used for in vitro transcription
(0.03 MB DOC)

Figure S1 Secondary structures of Eph/ephrin 3'UTRs as predicted by Mfold. Shown are only those parts of the 3'UTRs that contain HuR binding sites, prediction of secondary structure was applied on the full length 3'UTRs
(1.41 MB DOC)
