# Supplementary material for: Comparative 3'UTR Analysis Allows Identification of Regulatory Clusters that Drive Eph/ephrin Expression in Cancer Cell Lines
Source: PLoS One. 2008 Jul 23;3(7):e2780. doi: 10.1371/journal.pone.0002780 (PMC2474680; doi:10.1371/journal.pone.0002780)
Supplement: Table S1 — Lengths of Eph/ephrin 3'UTRs and accession numbers of Eph/ephrin mRNAs (0.04 MB DOC) [file pone.0002780.s001.doc]

| Accession | Gene | Length of 3’UTR [nt] | Length of alternative 3’UTR |
| --- | --- | --- | --- |
| BC002046 | EfnA1 | 756 |  |
| BC048697 | EfnA2 | 903 |  |
| AW123794, AK020438, NM_010108 | EfnA3 | 954 |  |
| BB472066, NM_007910, BE944428, AK012195 | EfnA4 | 897 | 552 |
| BC040218 | EfnA5 | 2594 |  |
| BU755816, NM_010110 | EfnB1 | 1465 | 539 |
| NM_010111, BC057009 | EfnB2 | 3149 | 2703 |
| BC052001 | EfnB3 | 1735 |  |
| BC071215 | EphA1 | 284 |  |
| BE986732, NM_010139 | EphA2 | 865 |  |
| BC093483, AK084921, BB482381 | EphA3 | 2599 | 317 |
| AV339884, AK147698 | EphA4 | 3310 |  |
| U07357 | EphA5 | 1252 |  |
| NM_007938 | EphA6 | 537 |  |
| NM_010141, BC026153 | EphA7 | 1075 | 390 |
| NM_007939 | EphA8 | 1629 |  |
| NM_173447 | EphB1 | 1358 |  |
| BC043088 | EphB2 | 1714 |  |
| NM_010143 | EphB3 | 788 |  |
| CJ189485, AK046884 | EphB4 | 878 |  |
| AK163823, CO043711 | EphB6 | 204 |  |
