# Supplementary material for: Comparative 3'UTR Analysis Allows Identification of Regulatory Clusters that Drive Eph/ephrin Expression in Cancer Cell Lines
Source: PLoS One. 2008 Jul 23;3(7):e2780. doi: 10.1371/journal.pone.0002780 (PMC2474680; doi:10.1371/journal.pone.0002780)
Supplement: Table S2 — Positions of CPEs, AREs, HuR binding sites and poly(A) hexamers in the Eph/ephrin 3'UTRs (0.05 MB DOC) [file pone.0002780.s002.doc]

|  | CPE  UUUUAU/  UUUUAAU | ARE  AUUUA | HuR binding site  NNUUNNUUU | AAUAAA/  AUUAAA |
| --- | --- | --- | --- | --- |
| EfnA1 | 748  708 | - | 704  683 | 734 |
| EfnA2 | 700 | 776  696  275  270 | 841-843  776  745-746  696-697  656  266-271  260-261 | 833 |
| EfnA3 | - | - | - | 935 |
| EfnA4 | 499 | - | - | 528 |
| EfnA5 | - | 2213  1470  1433  1388 | 2250  2209  2199  1825  1352  882-889  823-829  644-652  635-643  603  121  92 | - |
| EfnB1 | - | 99 | 1433  1338-1339  543-544  411-412  374-379  100 | - |
| EfnB2 | 3022  2993  2672 | 2921  2803  1765  1365  1046 | 3131  3017-3019  2984-2989  2921-2926  2744  1932  1863-1871  1710-1711  1219-1226  723 | 3124  2678 |
| EfnB3 | 1693 | - | 1571 | - |
| EphA1 | - | - | 156 | 262 |
| EphA2 | 799  746 | 805  81 | 791-795  771  755-758  741-742  508  314  77-81 | 841 |
| EphA3 | 2558 | 2550  2486  2367  2354  2065  1913  904  879  665  650  616  439 | 2482-2487  2452  2367  2350  2162  2117-2123  2083  2065-2066  1142  875  861  758  666  640  616-620  530  405-410  201  190  146-147 | 2565  298 |
| EphA4 | 3217  3141 | 3203  3037  2959  2331  2225  1127  814 | 3203  3157  3135-3141  2959-2960  2684-2685  1329  1123  923  70-75 | 3283 |
| EphA5 | 1209 | 914  595  573  484  58 | 906-910  784  756  595  573-574  57-58 | 1227 |
| EphA6 | 478 | - | 470-474  431-432 | 531 |
| EphA7 | 278 | 315  181 | - | 340 |
| EphA8 | - | - | 1131  1108 | 1604 |
| EphB1 | 1309  1270 | - | 1273  641-646  636  398-403 | 1323 |
| EphB2 | - | - | 1048  903  890  825 | 1693 |
| EphB3 | - | - | - | 770 |
| EphB4 | 845 | 823 | 838-841  688  524-525 | 853 |
| EphB6 | - | - | - | 176 |
