# Supplementary material for: Comparative 3'UTR Analysis Allows Identification of Regulatory Clusters that Drive Eph/ephrin Expression in Cancer Cell Lines
Source: PLoS One. 2008 Jul 23;3(7):e2780. doi: 10.1371/journal.pone.0002780 (PMC2474680; doi:10.1371/journal.pone.0002780)
Supplement: Table S3 — Primers used for Real Time RT-PCR (0.03 MB DOC) [file pone.0002780.s003.doc]

Table S3. Primers (5’→3’) Real Time RT-PCR

| experiment | Forward | Reverse |
| --- | --- | --- |
| RealTime  GAPDH | ccacccatggcaaattcc | tgggatttccattgatgacaag |
| Real Time  EfnA2 | ggtggaggtgagcatcaatga | cagcggcgccccata |
| Real Time  EphA2 | ccagttcagccaccacaaca | cccattctccatgtactcagtgat |
| Real Time  EphA4 | tggccgcacggaacat | ggacatgccaaaatcagacactt |
|  |  |  |
