# Supplementary material for: Comparative 3'UTR Analysis Allows Identification of Regulatory Clusters that Drive Eph/ephrin Expression in Cancer Cell Lines
Source: PLoS One. 2008 Jul 23;3(7):e2780. doi: 10.1371/journal.pone.0002780 (PMC2474680; doi:10.1371/journal.pone.0002780)
Supplement: Table S4 — Primers used for in vitro transcription (0.03 MB DOC) [file pone.0002780.s004.doc]

Table S4. Primers (5’→3’) in vitro transcription

| experiment | Forward | Reverse |
| --- | --- | --- |
| EfnA1 | ccaagcttctaatacgactcactatagggagagcccacatgtacagtgtctg | ttatgaatccaaaaacactgtata |
| EfnA2 | ccaagcttctaatacgactcactatagggagaggacgttgtcggtttatttctg | tcctaaaaagtccaccccac |
| EfnA4 | ccaagcttctaatacgactcactatagggagacttctgtgaagacggacttg | ttgaaggtttggtccaaacact |
| EfnB2 | ccaagcttctaatacgactcactatagggagacgttgtccaatttgtaagtaacac | ctgctcggatctcatttctg |
| EfnB3 | ccaagcttctaatacgactcactatagggagacgctctttagtgtgcagtgg | ttgttgttgttcaaatttcccttttac |
| EphA1 | ccaagcttctaatacgactcactatagggagaggtacaagtgaaggggactg | ggaagagatgaaaactcaactcttg |
| EphA2 | ccaagcttctaatacgactcactatagggagatgtcgctgtcagtgttacag | ttccagagcagaaataagtcat |
| EphA3 | ccaagcttctaatacgactcactatagggagagttgcacaatgtagatggcc | gatctgtaaaaatattttcattatgtttat |
| EphA4 | ccaagcttctaatacgactcactatagggagagaacaatcttttgagttagcca | gcaaactctttgcatttagtttg |
| EphA5 | ccaagcttctaatacgactcactatagggagaaattcaggcagagggtactc | ctgtcttagtataaaactctctgtg |
| EphA6 | ccaagcttctaatacgactcactatagggagatttgtgatcgagtagctcacg | cattgtatcaacatcaggcacc |
| EphA7 | ccaagcttctaatacgactcactatagggagaacatcccaacaccttgagct | gattttaacaataagatcatgatttttatttg |
| EphB1 | ccaagcttctaatacgactcactatagggagatgcatcccttcctgaaacct | aacccagacatgcttctgctc |
| EphA2 antisense | tgtcgctgtcagtgttacag | ccaagcttctaatacgactcactatagggagattccagagcagaaataagtcat |
