# Supplementary figures and images for: Comparative 3'UTR Analysis Allows Identification of Regulatory Clusters that Drive Eph/ephrin Expression in Cancer Cell Lines
Source: PLoS One. 2008 Jul 23;3(7):e2780. doi: 10.1371/journal.pone.0002780 (PMC2474680; doi:10.1371/journal.pone.0002780)

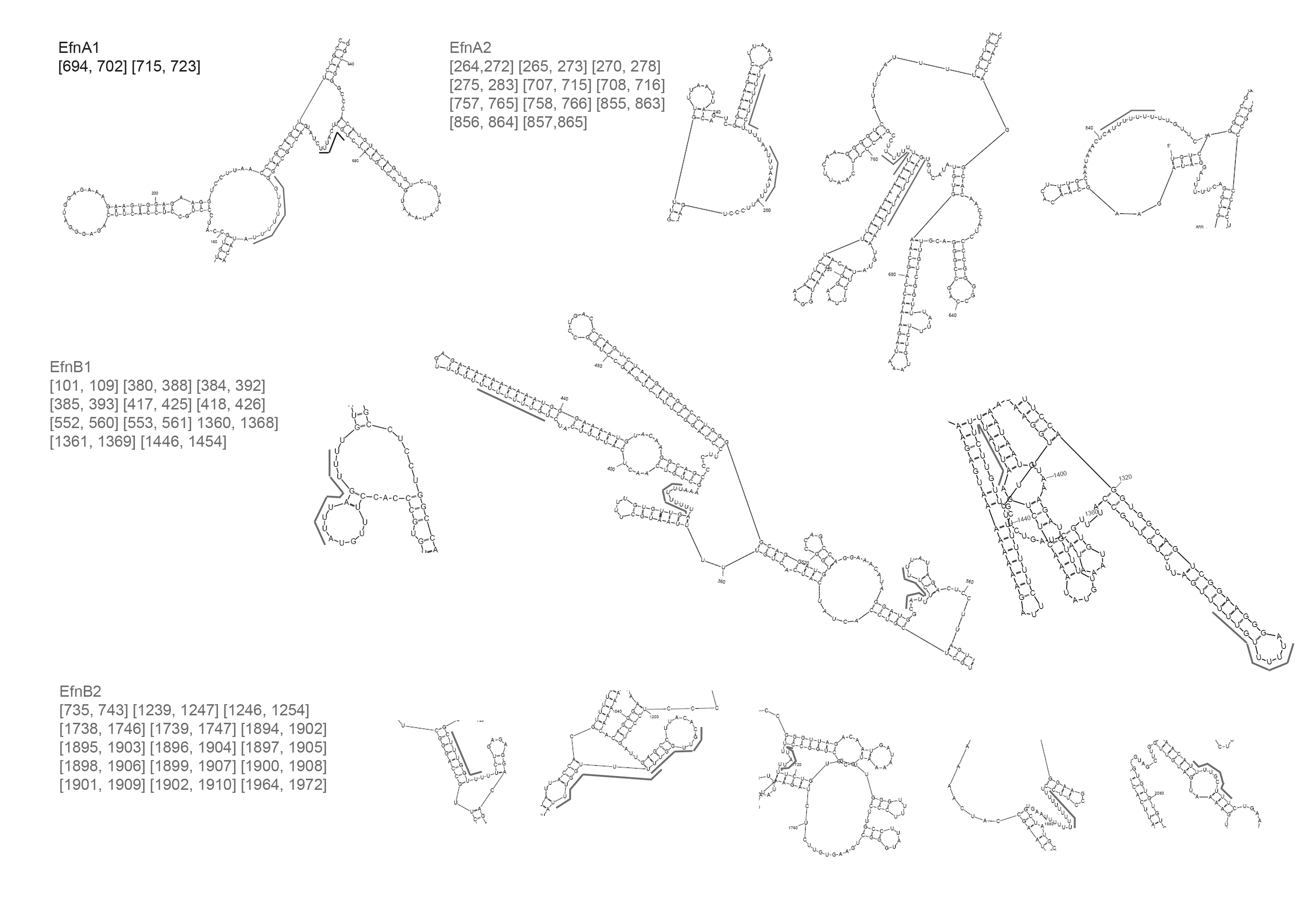

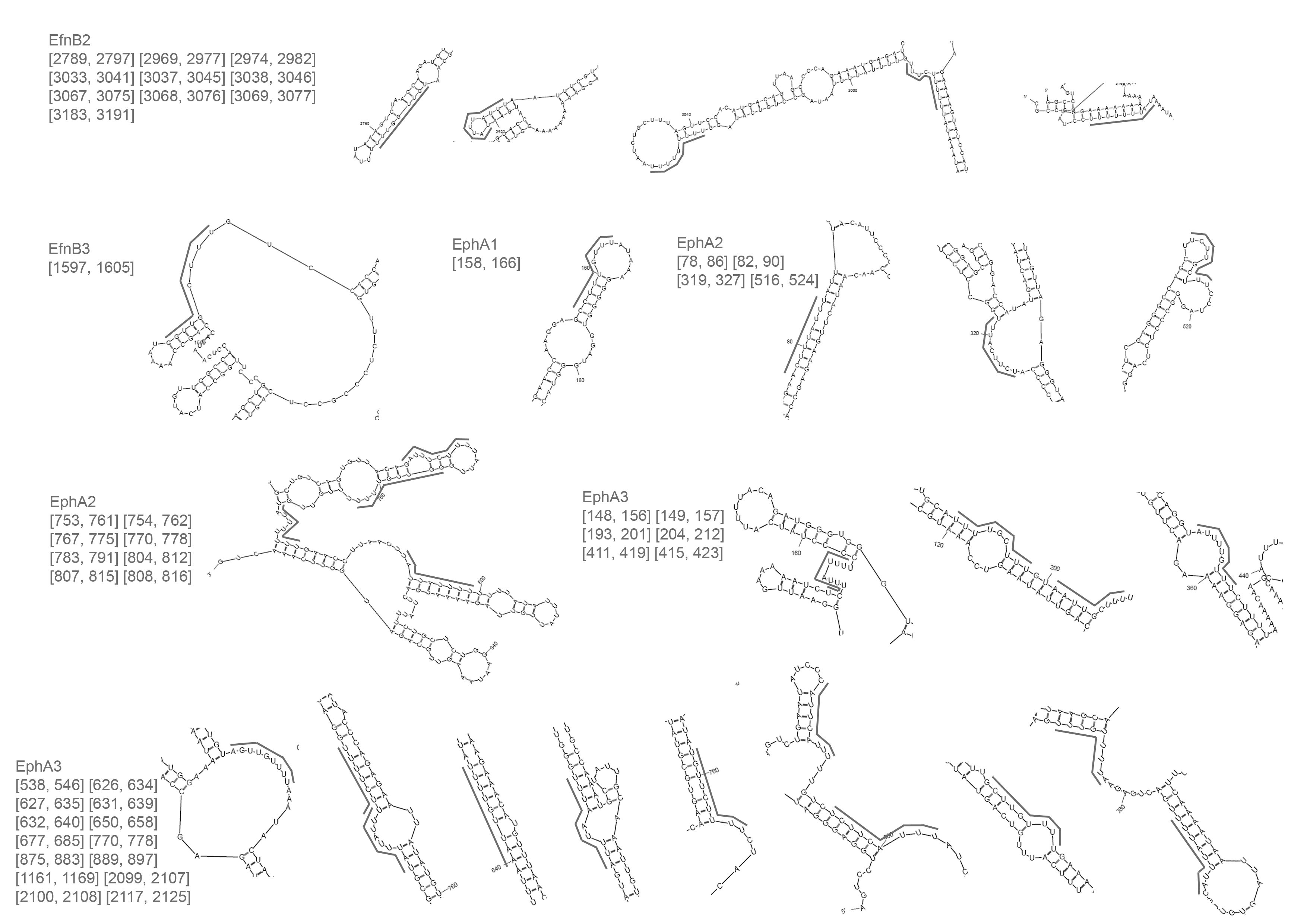

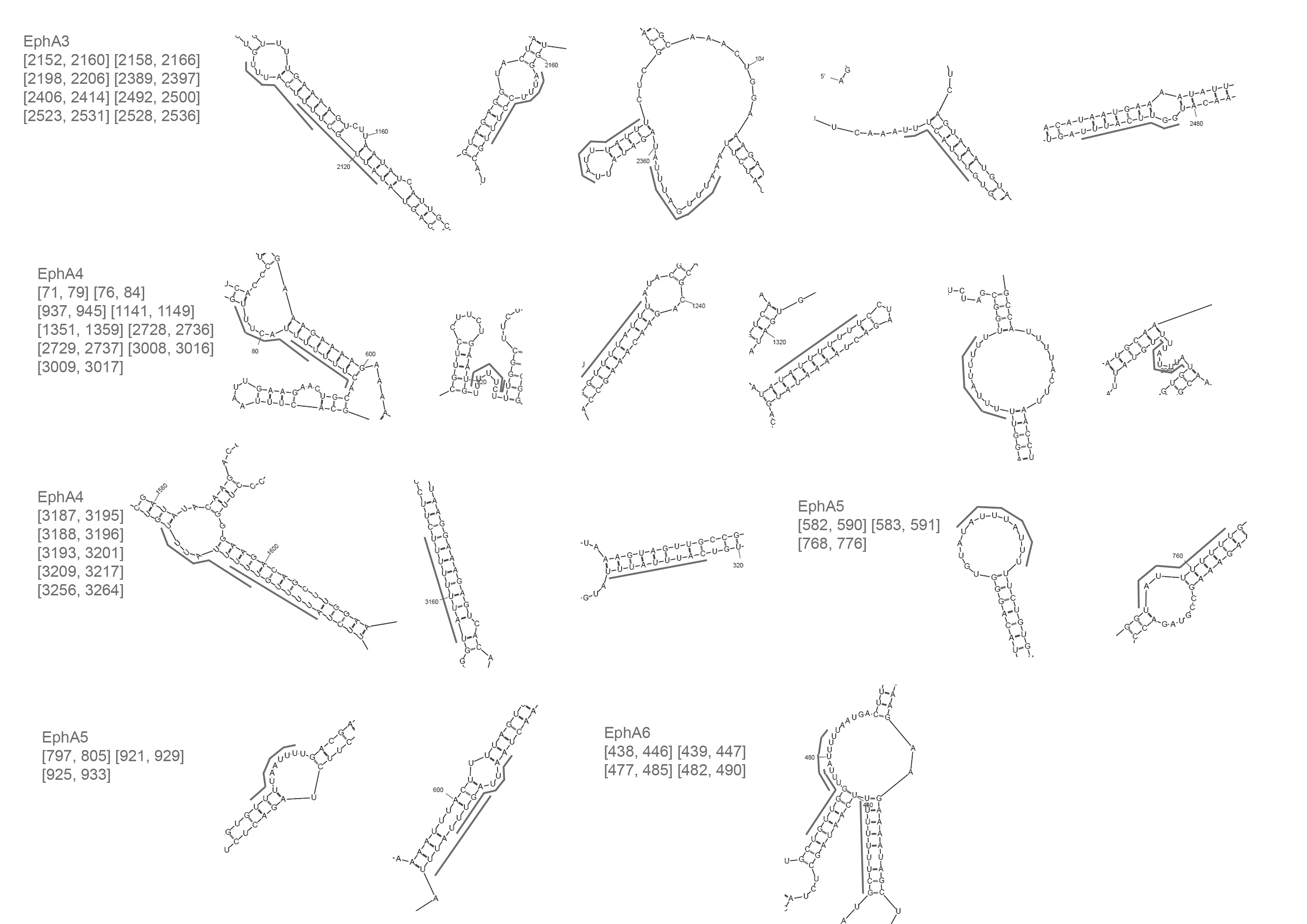

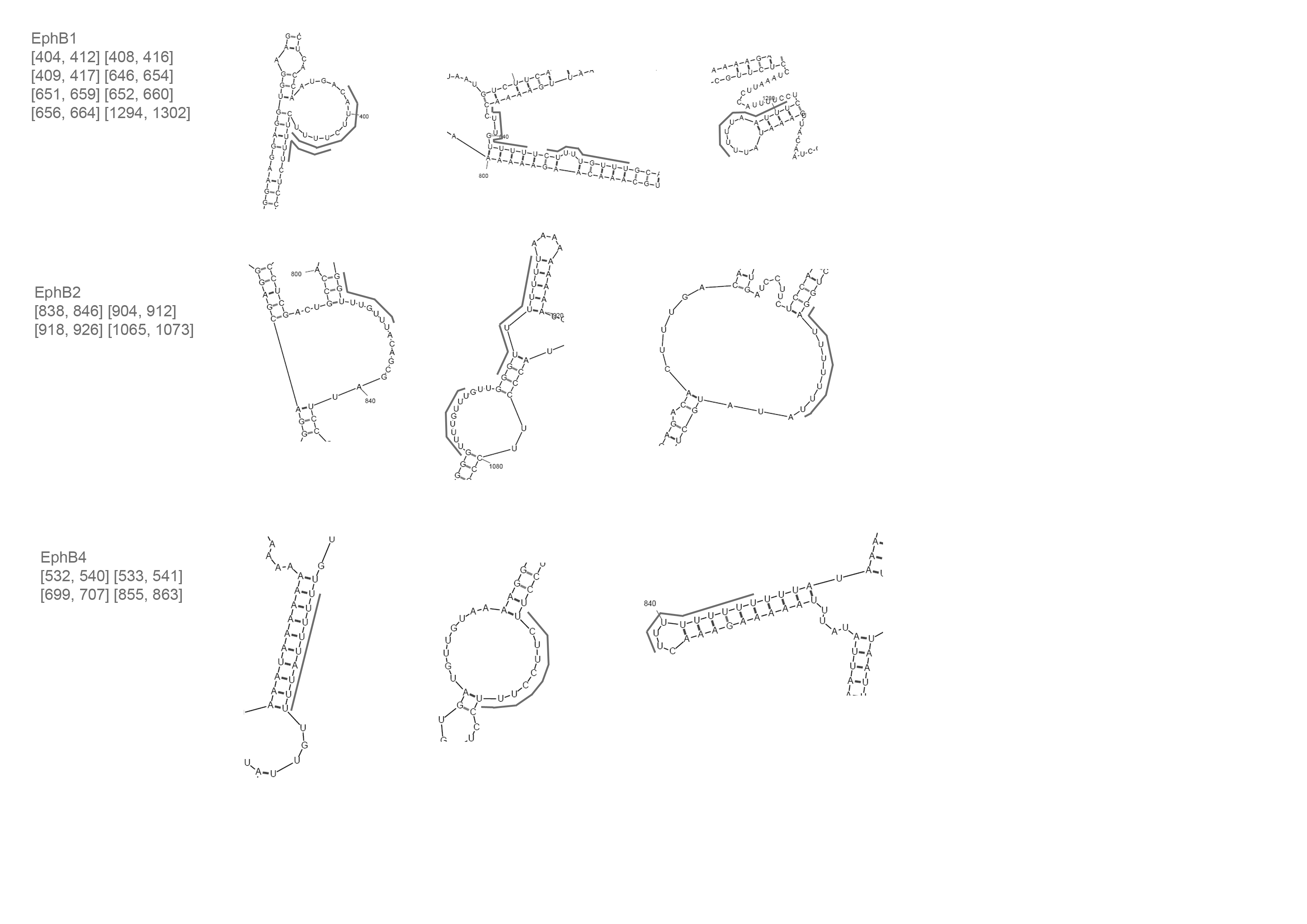

Supplement: Figure S1 — Secondary structures of Eph/ephrin 3'UTRs as predicted by Mfold. Shown are only those parts of the 3'UTRs that contain HuR binding sites, prediction of secondary structure was applied on the full length 3'UTRs (1.41 MB DOC) [file pone.0002780.s005.doc]
